# Use of the internet as a source of health information by Spanish adolescents

**DOI:** 10.1186/1472-6947-10-6

**Published:** 2010-01-29

**Authors:** Jaime Jiménez-Pernett, Antontio Olry de Labry-Lima, Clara Bermúdez-Tamayo, Jose Francisco García-Gutiérrez, Maria del Carmen Salcedo-Sánchez

**Affiliations:** 1 Andalusian School of Public Health, Granada, Spain; 2 CIBER en Epidemiología y Salud Pública (CIBERESP), Barcelona, Spain; 3 Alto Guadalquivir Hospital, Andujar, Spain

## Abstract

**Background:**

The Internet is a fundamental part of the daily life of adolescents, they consider it as a safe and confidential source of information on health matters. The aims is to describe the experience of Spanish adolescents searching for health information on the Internet.

**Methods:**

A cross-sectional study of 811 school-age adolescents in Granada was carried out. An adapted and piloted questionnaire was used which was controlled by trained personnel. Sociodemographic and health variables were included together with those concerning the conditions governing access to and use of information and communication technologies (ICT).

**Results:**

811 adolescents were surveyed (99.38% response rate), mean age was 17 years old. Of these, 88% used the Internet; 57.5% used it on a daily or weekly basis and 38.7% used it occasionally. Nearly half the sample group (55.7%) stated that they used the Internet to search for health-related information. The main problems reported in the search for e-health were the ignorance of good web pages (54.8%) and the lack of confidence or search skills (23.2%).

**Conclusions:**

In conclusion, it seems plausible to claim that websites designed and managed by health services should have a predominant position among interventions specifically addressed to young people.

## Background

The Internet is increasingly becoming part of our lives, its use being centred on the younger age-group (90.3% of citizens between 15 and 24 and 78.3% between 25 and 34 years of age are Internet users) [1].

The Internet is a fundamental part of the daily life of adolescents; they maintain permanent contact with their friends, form friendships with people who share their interests and hobbies and widen their knowledge [2,3]. As a consequence, this phenomenon has turned the Internet into one of the main means of communication between adolescents and is the reason why the younger age-group must be trained in the correct use of the Internet in relation to health information [4]. There are many reasons why the young are prompted to use the Internet, mainly because it is fast and easy and because it provides a great deal of information [5].

This is a particularly important aspect owing to the fact that adolescents find it hard to access traditional health services; the Internet also provides them with a confidential and safe source of health information [6]. The environment in which patients consume medical and health information has changed dramatically during the past decade. Rapid diffusion of Internet technology within the public sphere has placed an unprecedented amount of health information within reach of general consumers [7,8]. For adolescents, the internet is an integral part of their world, making e-Healthcare not only a reasonable extension of technology but an expectation [9].

In view of all this, this survey aims to describe the experience of adolescents in Andalusia (Spain) searching for health information on the Internet.

## Methods

A cross-sectional survey was carried out on adolescents in their post-compulsory schooling (or 11^th ^and 12^th ^grades in North America) in Andalusian high schools (Spain).

A calculation of sample size for a prevalence of 50%, with a 5% accuracy level and 95% confidence level was carried out, resulting in a total of 700 adolescents; in order to avoid any possible losses, calculation of the sample size was increased by 15%. The sample was selected by equiprobability single-stage cluster sampling. From among 42 secondary education schools providing post-compulsory schooling in Granada (Spain), nine were selected by simple random sampling, subsequently all the pupils attending the selected school were included.

### Variables

1) **Sociodemographic and health variables**: age, sex, perceived health and the number of visits to the doctor over the previous year. 2) **Variables related to the conditions governing the access to and use of information and communication technologies (ICT)**: Use of the Internet, frequency of use, main use of the Internet (games, travel, shopping, e-Learning, others), who the results are aimed at, opinion of the Internet as a good source of health information and the use of other sources of health information. Three multi-answer questions were formulated in order to obtain the greatest amount of information available to identify Internet access points, sources for health information searches and health subjects searched.

### Instruments

The information was gathered using a structured questionnaire with 22 items. This self-administered questionnaire was adapted and piloted [10]. from a Kaiser Family Foundation study [4]. Their instrument was designed and analyzed by staff of the organization, in consultation with International Communications Research.

### Procedure

Prior to gathering the information, a pilot test was carried out on a sample of 50 adolescents in order to determine the proper functioning of the questionnaire; the data collected was not included in the analyses carried out [10].

First, the research protocol had to be approved by the *Research and Ethics Committee *of the *Progress and Health Foundation *of the *Andalusian Regional Ministry of Health*. Subsequently, contact was made with those in charge of each school and after obtaining approval of the heads and teaching staff of schools, the adolescents were asked to take part in the survey. Informed consent on the part of students was verbally requested.

The questionnaires were controlled by personnel specifically trained for this survey who had good communication skills. In addition, no information was collected that could facilitate the identification of respondents.

### Statistical analysis

A descriptive analysis of the sample group was carried out using the standard frequencies, percentages for qualitative variables and means and deviations for quantitative variables. Eventually, a bivariate analysis was carried out by means of the chi-square test. The analysis was carried out using the statistical programme SPSS 11.5.

## Results

A total of 811 adolescents were studied, 46% of them were men, 17 years old was the average age of sample (none of the youngsters refused to take part in the survey). An average of 2 visits to the doctor were made over the previous year and 9.3% reported an average, bad or very bad self-perceived state of health compared with 26.3% who considered their health to be very good.

As far as the access and use of information and communication technology variables are concerned, 88% used the Internet, 57.5% used it on a daily or weekly basis and 38.7% used it occasionally. Only 7.8% of the adolescents thought that more information would improve their health.

Nearly half the sample (55.7%) stated that they used the Internet to search for health information, 19.4% used it on a daily or weekly basis compared with 25.7% who used it occasionally during the year/not at all. The main problems reported when searching for e-health was a lack of knowledge of good web pages (54.8%) and a lack of confidence or search skills (23.2%). Among those who used the Internet, 82% stated that it helped them obtain more health information and 54.9% answered that the Internet helped them obtain better health information (Table 1).

**Table 1:** Summary of the main variables (n = 811).

		N (%)
Gender	Male	373 (46%),
	Female	438 (54,1%)

Perceived health	Very good	213 (26.3%)
	Good	521 (64.4%)
	Not so good-Bad/very bad	75 (9.3%)

General use of the Internet	No	97 (12%)
	Yes	713 (88%)

Frequency of use of the Internet	Daily/A few times a week	410 (57.5%)
	A few times a month	276 (38.7%)
	A few times a year-Never	27 (3.8%)

Do you search for health information on the Internet?	No	359 (44.3%)
	Yes	451 (55.7%)

Frequency of health information searches on the Internet	Daily/A few times a week	88 (19.4%)
	A few times a month	249 (54.8%)
	A few times a year - Never	117 (25.7%)

Do you think your QOL would improve if you had more health information?	No	63(7.8%)
	Yes	580(72%)
	Do not know	162(20.1%)

Problems when looking for health information on the Internet (choose one)	Do not know good sites	239 (54.8%)
	Lack of search skills	101 (23.2%)
	No Internet at home	24 (5.5%)
	Not interested in the subject	15 (3.4%)
	No confidence/no search/Other	57 (13.1%)

What sources do you use to find health information? (mark all)	Family and friends	456 (56.4%)
	Magazines, newspapers	357 (44.2%)
	Books/medical encyclopaedias	179 (22.2%)
	Television	348 (43.1%)
	Radio	29 (3.6%)
	Internet	424 (52.5%)
	Doctors, nurses	488 (60.4%)
	Chemists	177 (21.9%)
	None	54 (6.7%)

Why do you search health information on the Internet (choose one)	Easy	158 (36.9%)
	Lots of information	93 (21.7%)
	Fast information	63 (14.7%)
	Confidential search	45 (10.5%)
	See different opinions	34 (7.9%)
	Free information	24 (5.6%)
	Other	11 (2.6%)

		**Median (DS)**

Age (in years)		17 (1.01)

Visits to the doctor during the previous year		2 (3.92)

As far as the multi-answer variables are concerned (Table 2), the three most popular sources for obtaining information on health were: family and friends (56.4%), doctors and nurses (21.9%) and the Internet (60.4%). The adolescents reported accessing the Internet mainly from their homes (93.5%), followed by school centres (28.4%) and cybercafés (14%). They mainly used the Internet for leisure pursuits, music and games (90%), school work (87%) and chats and forums (79.7%). And 97.6% of respondents obtained their health information through a search engine (mainly Google), followed by newspapers (50.1%) and out-links in the websites they visited (45.5%). Regarding the intended users of the information, 95.6% of the subjects stated that the information was for personal use. The main reasons for searching health information on the Internet were: easy of use (36.9%), lots of information (21.7%) and speed (14.7%). The main health topics looked up on the Internet were: Self-image, beauty and wellness (56.5%), fitness and physical activity (53%) and piercing and tattoos (44.6%) (Table 3).

**Table 2:** Summary of the main variables (n = 811).

		N (%)
Where do you usually access the Internet? (Multiple choice)	Home	666 (93,5%)
	School	202 (28,4%)
	Cultural or leisure centres	23 (3,2%)
	Cybercafé	100 (14%)
	Mobile phone	26 (3,7%)
	Other places	52 (7,3%)

What do you usually use the Internet for? (Multiple choice)	Leisure, music, games	642 (90%)
	School work	620 (87%)
	Searching for information	564 (79,1%)
	Shopping	47 (6,6%)
	Courses	32 (4,5%)
	E-mail	519 (72,8%)
	Chat (e.g. messenger), forums	568 (79,7%)
	Others	49 (6,9%)

Why do you look for health information in the internet? (Multiple choice)	It is free	24 (5,6%)
	It is quick	63 (14,7%)
	It is confidential	45 (10,5%)
	It is easy	158 (36,9%)
	There are a lot of information	93 (21,7%)
	To have different opinions	34 (7,9%)
	Others	11 (2,6%)

¿How do you look for health information? (Multiple choice)	Internet searcher	444 (97,6%)
	Family/friends	167 (36,7%)
	Physicians	39 (8,6%)
	News paper, media	228 (50,1%)
	Links of websites	207 (45,5%)
	Others	17 (3,7%)

¿Whom do you look health information for? (Multiple choice)	Yourself	434 (95,6%)
	Family	182 (40%)
	Classmates or friends	142 (31,2%)
	Others	28 (6,2%)

**Table 3:** Twelve main health topics looked up on the Internet (n = 451*).

Health topic	N(%)
Body image, beauty and wellness	255 (56,5%)

Fitness and physical activity	239 (53%)

Piercing and tattoos	201 (44,6%)

Contraception	185 (41%)

Nutrition and diet	184 (40,8%)

Sexual behavior	183 (40,6%)

Drugs	165 (36,6%)

Sexually Transmitted Diseases	147 (32,6%)

Skin conditions	144 (31,9%)

Tobacco and alcohol	119 (26,4%)

Eating disorders	103 (22,8%)

Physical development	80 (17,7%)

* Respondents that have searched for health information on the Internet

The bivariant analysis showed that those adolescents who used the Internet on a daily basis were most likely to search for health information (OR: 1.49; IC95% 2.02-1.01). Concerning the information sources, those who searched for health information on the Internet demonstrated, with a significant statistical difference, a greater probability of consulting doctors (60.8% vs. 51.1%), magazines (49.9% vs. 37.7%), books (27.9% vs. 14.9%) and, in contrast, a lesser degree of probability of consulting none (1.6% vs. 13.6%).

## Discussion

Moreover, this survey opens a small window through which we can begin to study the opinion that Spanish adolescents hold of the Internet and how they relate to it. Thus it is demonstrated that the young mainly use the Internet for leisure pursuits, to search for information and to communicate with their peers.

The percentage of adolescents looking for health information on the Internet reflected in this study is within the range described in existing literature (40%-73.4%) [4,11-16]. This result is considerably remarkable since most research articles related to this topic have been conducted on adolescents who were native speakers of English and there are no articles on Spanish adolescents.

Literature on the topic reflects, similarly to this study, that there is a statistically significant difference between adolescents who browse the Internet more intensively and those who do it less frequently, the former being more likely to search for information on health matters than the latter [15-17].^. ^Moreover, health topics most frequently searched by respondents correspond with those identified in previous studies: body image, exercise and fitness, diets and nutrition, sexual information and sexually transmitted diseases [9,11,18,19].

This study has several limitations which should be taken into account in order to analyse the results. Firstly, although the aim of this survey was not to determine or acknowledge any gaps or shortcomings in IT training or computer literacy, the existence of these could bias the results shown. On the other hand, a cluster sample was performed, which makes it impossible to know beforehand the number of questionnaires, however, in Spain there are a limited number of students per classroom (nearly 30).

One significant result was that when asked which pages they used most to obtain health information all the adolescents surveyed replied that they accessed the information through Google-type search engines. It seems to show clearly that these mechanisms prevail mainly because they are easy, accessible, fast and contain a great deal of information [20].^. ^Nevertheless, it is significant that these kind of web sites works by means of algorithms and some web pages are more likely to be among those recommended than others. This is especially important because these pages are usually sponsored by the private sector, which could influence young people [21], and because of the quality differences reported in their content [22]. Training adolescents in the correct use of the online information sources is a key element in optimizing interaction with the web [23].

## Conclusions

From the point of view of public health, these results represent an opportunity for health services. Hence, it is necessary to design websites more adequate for adolescents needs that may provide them with reliable and confidential information. In this regard, literature depicts that children and teenagers tend to believe that "if you can find it online then it must be true" [24]. In conclusion, it seems plausible to claim that websites designed and managed by health services should have a predominant position among interventions specifically addressed to young people.

## Competing interests

The authors declare that they have no competing interests.

## Authors' contributions

AOLL, JJP and CBT drafted the manuscript and performed the statistical analysis. JJP, MCSS and JFGG participated in the design and of the study. JJP and JFGG conceived the study, participated in its design and coordination and helped to draft the manuscript. All authors read and approved the final manuscript.

## Pre-publication history

The pre-publication history for this paper can be accessed here:

http://www.biomedcentral.com/1472-6947/10/6/prepub

## References

[B1] Instituto Nacional de estadística Survey on the Equipment and Use of ICT http://www.ine.es/[Accessed: 2010-01-20]

[B2] Punamäki RL Wallenius M Nygard CH Saarni L Rimpelä A Use of information and communication technology (ICT) and perceived health in adolescence: The role of sleeping habits and waking-time tiredness J Adolesc 2007 30 4 569 85 10.1016/j.adolescence.2006.07.004 16979753

[B3] Buckingham D Lievrouw LA The electronic generation? Children and new media Handbook of new media: Social shaping and consequences of ICTs 2002 7 Livingstone, London: Sage 7 89

[B4] Rideout V Generation Rx: How young people use the Internet for Health information Kaiser Family Foundation Survey 2001 http://www.kff.org/entmedia/loader.cfm?url=/commonspot/security/getfile.cfm&PageID=13719 [Accessed: 2010-01-20]

[B5] Kanuga M Rosenfeld WD Adolescent sexuality and the internet: the good, the bad, and the URL J Pediatr Adolesc Gynecol 2004 17 117 124 10.1016/j.jpag.2004.01.015 15050988

[B6] Gray NJ Klein JD Noyce PR Sesselberg TS Cantrill JA Health information-seeking behaviour in adolescence: the place of the internet Soc Sci Med 2005 60 1467 78 10.1016/j.socscimed.2004.08.010 15652680

[B7] Rice RE The Internet and health communication: a framework of experiences The Internet and Health Communication: Experiences and Expectations 2001 Thousand Oaks, Calif: Sage Publications 5 46

[B8] Hesse BW Nelson DE Kreps GL Croyle RT Arora N Rimer BK Trust and Sources of Health Information The Impact of the Internet and Its Implications for Health Care Providers: Findings From the First Health Information National Trends Survey Arch Intern Med 2005 165 2618 2624 10.1001/archinte.165.22.2618 16344419

[B9] Kell B The strengths and limitations of the internet as a health information resource for adolescents J Adolesc Health 2009 4 s20 10.1016/j.jadohealth.2008.10.057

[B10] Jiménez-Pernett J Salcedo-Sánchez MC García-Gutiérrez JF Necesidades y expectativas de información sobre salud en Internet para adolescentes: Una prueba piloto Revista eSalud 2007 3 10 http://www.revistaesalud.com [Electronic journal]. Accessed: 2010-01-20]

[B11] Gould MS Harris Munfakh JL Lubell K Kleinman M Parker S Seeking help from the Internet during adolescence J Am Acad Child Psy 2002 41 1182 10.1097/00004583-200210000-00007 12364839

[B12] Bleakley A Merzel CR VanDevanter NL Messeri P Computer access and internet use among urban youths Am J Public Health 2004 94 744 10.2105/AJPH.94.5.744 15117693 PMC1448330

[B13] Gray NJ The Internet: A window on adolescent health literacy J Adolescent Health 2005 37 243 e1 e7 10.1016/j.jadohealth.2004.08.023 16109345

[B14] Borzekowski DL Rickert VI Adolescents, the Internet, and health: issues of access and content J App Dev Psychol 2001 22 49 59 10.1016/S0193-3973(00)00065-4

[B15] Ogan CL Ozaka M Groshek J Embedding the Internet in the lives of college students. Online and Offline behaviour Soc Sci Comput Rev 2008 26 170 177 10.1177/0894439307306129

[B16] Hufken V Deutschmann M Baehring T Scherbaum W [Use of the internet for health care information: results from a national telephone survey] Soz Praventivmed 2004 49 6 381 90 10.1007/s00038-004-3149-0 15669438

[B17] Lenhart A Madden M Macgill AR Smith A Teens and social media. Pew Internet and American Life Project report, 2007 [online] http://www.pewinternet.org [Accessed: 2010-01-20]

[B18] Skinner H Biscope S Poland B Goldberg E How adolescents use technology for health information: implications for health professionals from focus group studies J Med Internet Res 2003 5 e32 10.2196/jmir.5.4.e32 14713660 PMC1550577

[B19] Borzekowski DL Fobil JN Asante KO Online Access by Adolescents in Accra: Ghanaian Teens' Use of the Internet for Health Information Developmental Psychology 2006 42 450 458 10.1037/0012-1649.42.3.450 16756437

[B20] Boyer C Geissbuhler A Health and the Internet for all Int J Med Inform 2006 75 1 3 10.1016/j.ijmedinf.2005.07.039 16325462

[B21] Miller EA West DM Characteristics associated with use of public and private web sites as sources of health care information Med Care 2007 45 245 251 10.1097/01.mlr.0000244509.60556.49 17304082

[B22] Babio G Bermúdez Tamayo C García JF Márquez S Selección y evaluación de sitios web dirigidos a pacientes Andalusian Agency for Health Technology Assessment 2006 http://www.hvn.es/invest_calid_docencia/bibliotecas/publicaciones/archivos/doc_52.pdf [Accessed: 2009-01-26]

[B23] Truccolo I Bianchet K Dal Maso L Cognetti G De Paoli P Internet as a health education tool by Italian high school students Journal of the European Association for Health Information and Libraries 2009 5 http://www.eahil.net/journal/journal_2009_vol5_n2.pdf#page=15 [Electronic journal]

[B24] Bremer J The internet and children: advantages and disadvantages Child Adolesc Psychiatr Clin N Am 2005 14 3 405 28 viii 10.1016/j.chc.2005.02.003 15936666

